# Supplementary material for: Respiratory disease in workers handling cross-linked water-soluble acrylic acid polymer
Source: PLoS One. 2023 Apr 21;18(4):e0284837. doi: 10.1371/journal.pone.0284837 (PMC10120934; doi:10.1371/journal.pone.0284837)
Supplement: Attachment — Submitted filename: Response to Reviewers_L3_TABWCJ-8.docx [file pone.0284837.s001.docx]

February 10, 2023

Emily Chenette

Editor-in-Chief

*Plos One*

Dear Editor:

Thank you for allowing us to submit a revised draft of the manuscript entitled “Respiratory disease in workers handling cross-linked water-soluble acrylic acid polymer” for publication in *Plos One*. We appreciate the time and effort that you and the reviewers dedicated to providing feedback on our manuscript and are grateful for the insightful comments on and valuable improvements to our paper. We have uploaded a copy of the original manuscript marked with all the changes made during the revision process. The revisions are shown as track changes in the manuscript file. Also appended to this letter are point-by-point responses to the comments raised by the reviewers.

1. Please ensure that your manuscript meets PLOS ONE's style requirements, including those for file naming.

Response: Thank you for your remark. We have checked and confirmed that our manuscript meets these requirements.

2. We note that the grant information you provided in the ‘Funding Information’ and ‘Financial Disclosure’ sections do not match.

When you resubmit, please ensure that you provide the correct grant numbers for the awards you received for your study in the ‘Funding Information’ section.

Response: Per the editor's suggestion, we have corrected the appropriate sections. This study was not funded by a commercial company and was financially supported by a grant-in-aid from the Japan Organization of Occupational Health and Safety (Collaborative Research). There is no grant number in this research fund.

3. Your ethics statement should only appear in the Methods section of your manuscript. If your ethics statement is written in any section besides the Methods, please delete it from any other section.

Response: We have made the appropriate changes.

**Reviewers' Comments to the Authors:**

5. Review Comments to the Author

**Reviewer 1**

1) Were any blood investigations like ANA, RA factor, ACE levels done in these patients to exclude other kinds of ILDs?

Author response: Thank you for your questions. All of ANA, RA factor, ACE are negative.

2) Were these patients on any drugs/ receive any radiation in the past that could have lead to an ILD? Were these included in the questionnaire?

Author response: There were no drugs and radiation for all cases.

3) was a pre employment x ray, PFT done in these patients?

Author response: The pre-employment X-ray was normal and no PET was done.

4) Were these patients subjected to PFT to know whether they have a restriction, obstruction or mixed pattern?

Author response: The restriction pattern is 5 and mixed pattern 1, normal 2.

5) Did any of these patients develop any skin allergies in addition to the respiratory problems?

Author response: The patients have no skin allergies.

6) What were the shift hours of the patient like?

Author response: They work for eight hours.

What kind of protective masks were they using?

Author response: Anti-dust exchangeable masks were used.

What was the kind of ventilation in the rooms that they were working in?

Author response: A device for exhausting dust was present in the rooms.

What the smoking index in the patients and was there any correlation with the progression?

Author response: The smoking index was 200 for Case 1, 460 for Case 2, 260 for Case 3, and 500 for Case 4.

Do you think these could have also made an impact on the severity of their problems?

Author response: Very high concentrations of dust may induce these problems of pulmonary and pleural diseases.

7) Were these patients better on their off days i. e. better on their non-working days?

Author response: These patients do not get better on their off days.

**Reviewer 2**

**Abstract**

Line 32- add space after above

Author response: Thank you for your comment. We have made the required change.

Line 37-change this disease’s to such disease

Author response: We have made the required change.

Line 38-change inhaling organic substances to cross-linked water-soluble acrylic acid polymer (other organic substances cause fibrosis, see line 52)

Author response: We have made the appropriate changes (New line 39).

**Introduction**

Line 48-add ‘incidences of’ after “after”

Author response: We have made the appropriate changes (New line 50).

**Figure and Figure Legends**

1. Legend to fig 1 seems incomplete. You may want to explain the tasks here

Author response: The details of the work was described in detail (please refer to the legend of figure 1).

2. Is fig 2 a representative of all cases or just case 1 and you need to show more. Please highlight or point to the findings with arrows

Author response: Thank you for your insightful comment. We added case 2, a typical case similar to case 1, as figure 4. In figures 2 and 4, we inserted arrows and round arrows to indicate points. For figure 4, we added an explanatory note.

3. Fig 3 needs to be labeled and all the findings indicated in the legend needs to be indicated on the figures. Give subheading/ labels to each picture in the figure like in figure 4

Author response: Arrows or round arrows have been added to the figure. We labeled figures 3A, 3B, 3C, and 3D and inserted black arrows (large and small) and white arrows to indicate the findings. We have also added an explanatory note.

4. Figure legend should have same labels like 4A not 4a. Indicate the findings with arrows

Author response: We have newly labeled the figure as Fig. 5A and 5B, inserted white arrows to indicate the findings, and each explanatory note is the original.

5. Capitalize the subheading in the legend. Red circular arrow is blurred and demonstrate other findings with arrows

Author response: We deleted the red circular arrow and indicated findings using arrows and round arrows. The Explanatory note is the original.

6. Are both 6A and 6B interlobular septal thickening as mentioned in the legend. What do red circle indicate

Author response: We deleted the red circular arrow in figures 7A and 7B and indicated findings using arrows and round arrows. The Explanatory note is the original.

7. Figure 8 is unclear what are the two scans. What is the box for. What do the arrows indicate

Author response: The two scans are shown in new figures 9 and 11, with one scan per figure. We deleted the arrows and the explanatory note for the lesioned area is the original.

8. Carefully check each figure and legend to improve clarity.

Author response: We have rechecked figures and legends and made the required changes.

9. New figures 10, 12, and 13 are presented in order of magnification, and to make it easier to view the EW staining, it was presented with decreasing magnification.

We believe that the findings of this study are relevant to the scope of your journal and will be of interest to its readership. The manuscript has been carefully reviewed by an experienced editor whose first language is English and who specializes in editing papers written by scientists whose native language is not English.

We look forward to hearing from you at your earliest convenience.

Sincerely,

Takumi Kishimoto

Director of Research and Training Center for Asbestos-Related Diseases

1-10-25 Chikko Midorimachi, Minami-ku, Okayama 702-8055 Japan

Phone No: +81-86-262-9166

Fax No: +81-86-280-2828

Email Address: nakisimt@okayamah.johas.go.jp
